# Supplementary material for: Differentiation signals from glia are fine-tuned to set neuronal numbers during development
Source: eLife. 2022 Sep 12;11:e78092. doi: 10.7554/eLife.78092 (PMC9507125; doi:10.7554/eLife.78092)
Supplement: Supplementary file 1. [file elife-78092-supp1.docx]

**Supplementary File 1: Table 1 summarising the results from the glial-Gal4 screen (Figure 1 B,C, Figure 1- figure supplement 1B-N) to identify the glial type that regulates L5 development.**

| ***Glial-type > EGFR^DN^x2*** | **Elav+ cells in the proximal lamina** |
| --- | --- |
| Pan-glia | Absent or reduced |
| Perineurial glia | Present |
| Sub-perineurial glia | Present |
| Cortex glia | Present |
| Epithelial glia and Marginal glia (eg + mg) | Present |
| Wrapping glia and xg^outer^ (xg^O^) | Absent or reduced |
| xg^O^ +xg^inner^ | Absent or reduced |
| xg^O^ | Absent or reduced |
| xg^Ots^ | Absent or reduced |
